# Supplementary material for: Structural Characterization and In Vitro Anti-Inflammatory Activity of Polysaccharides Isolated from the Fruits of Rosa laevigata
Source: Int J Mol Sci. 2024 Feb 9;25(4):2133. doi: 10.3390/ijms25042133 (PMC10888661; doi:10.3390/ijms25042133)
Supplement: Supplementary file 1 [file ijms-25-02133-s001.zip › ijms-2796829-supplementary.pdf]

Structural characterization and *in vitro* anti-inflammatory activity test of polysaccharides isolated from the fruits of *Rosa laevigata*

Song Peng [a,b], Pengfei Gu [c], Ningning Mao [a,b], Lin Yu [a,b], Tianyu Zhu [a,b], Jin He [a,b], Yang Yang [a,b], Zhenguang Liu [a,b], Deyun Wang [a,b,*]

[a] Institute of Traditional Chinese Veterinary Medicine, College of Veterinary Medicine, Nanjing Agricultural University, Nanjing 210095, PR China.

[b] MOE Joint International Research Laboratory of Animal Health and Food Safety, College of Veterinary Medicine, Nanjing Agricultural University, Nanjing 210095, PR China.

[c] College of Traditional Chinese Veterinary Medicine, Hebei Agricultural University, Baoding, 071001, China.

* Corresponding authors.

E-mail addresses: dywang@njau.edu.cn

Supplementary Method

S1. HPAEC conditions

The sample extracts were analyzed by high-performance anion-exchange chromatography (HPAEC) on a CarboPac PA-20 anion-exchange column (3 by 150 mm; Dionex) using a pulsed amperometric detector (PAD; Dionex ICS 5000 system) by Sanshu Biotech. Co., LTD (Shanghai, China). Flow rate, 0.5 mL/min; injection volume, 5μL; solvent system A : (ddH_2O), solvent system B : (0.1M NaOH), solvent system C : (0.1M NaOH, 0.2M NaAc); gradient program, the volume ratio of solution A, B, C was 95:5:0 at 0 min, 85:5:10 at 26 min, 85:5:10 at 42 min, 60:0:40 at 42.1 min, 60:40:0 at 52 min, 95:5:0 at 52.1 min, 95:5:0 at 60 min.

S2. Methylation analysis of RLPa-2

Approximately 5 mg of the sample was dissolved in pure water and reacted with 1 mL of 1-cyclohexyl-2-morpholinoethylcarbodiimide methyl p-toluenesulfonate (CMC, 100 mg/mL) for 2 h. The product was mixed with 1 mL of imidazole (2 mol/L), and divided equally into two parts. Then, one part was added with 1 mL NaBH4 (30 mg/mL), and the other part was added with 1 mL NaBD4 (30 mg/mL), dialyzing and lyophilizing to acquire the reduzates, and the reduzates were methylated in DMSO/NaOH with CH_3I. After complete methylation, the permethylated products were hydrolyzed with TFA (2 mol/L) at 121°C for 1.5 h, reduced by $NaBD_4$ (1 mol/L) and acetylated with acetic anhydride for 2.5 h (100 °C). Finally, 500 μL of dichloromethane was added, vortexed, mixed, centrifuged, and repeated thrice. The

aqueous phase was discarded, the lower dichloromethane phase was taken, and the product was analyzed by gas chromatography-mass spectrometry (GC-MS) (Agilent 7890A-5977B; Agilent Technologies Inc., USA) for analysis, and high purity helium (split ratio 10:1) was used as the carrier gas with an injection volume of 1 μL. Mass spectrometry analysis was performed at the initial temperature of 140 ℃ for 2.0 min, and the temperature is increased to 230 ℃ by 3 ℃/min for 3 min. The scan mode was SCAN with a range from 30 to 600 m/z.

Supplementary Data

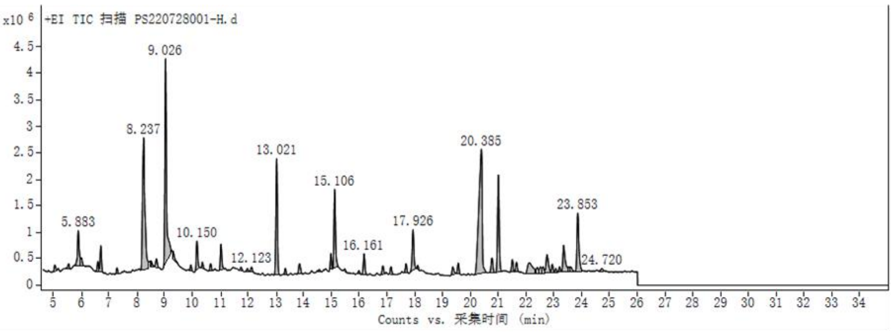

Figure S1. The total ion chromatogram of RLPa-2 polysaccharides was methylated.

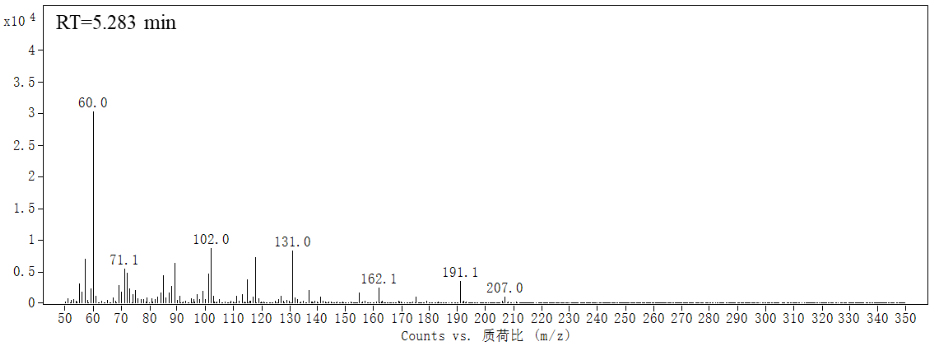
RT=5.283 min
x10 4
60. 0
71. 1
102. 0
131. 0
162. 1
191. 1
207. 0
Counts vs. 质荷比 (m/z)

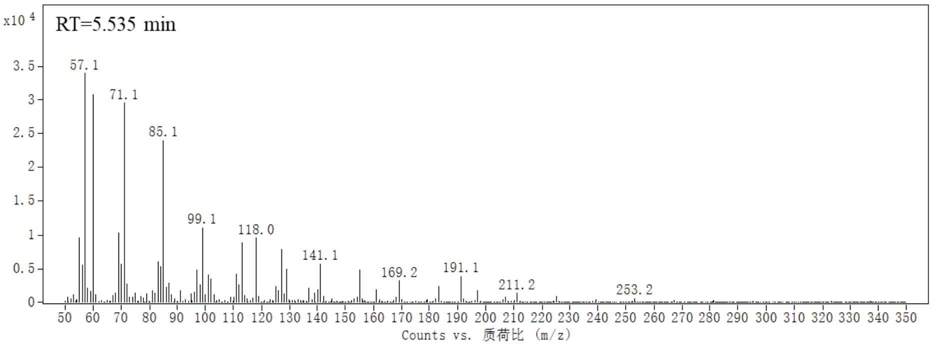
RT=5.535 min
x10 4
57. 1
71. 1
85. 1
99. 1
118. 0
141. 1
169. 2
191. 1
211. 2
253. 2
Counts vs. 质荷比 (m/z)

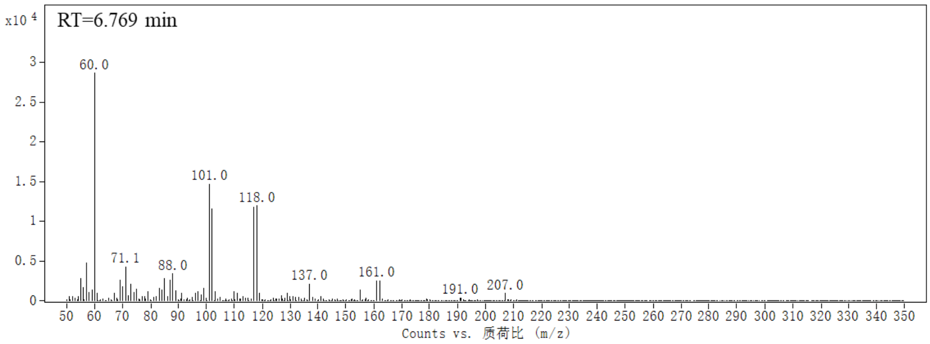
RT=6.769 min
x10 4
60. 0
101. 0
118. 0
71. 1
88. 0
137. 0
161. 0
191. 0
207. 0
Counts vs. 质荷比 (m/z)

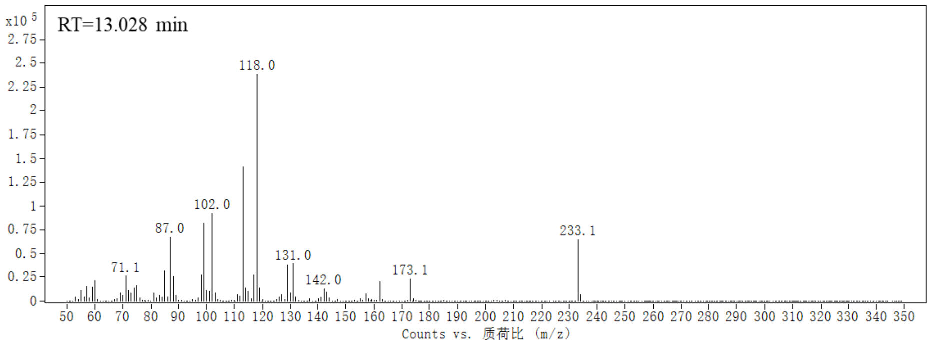
RT=13.028 min
x10 5
118. 0
102. 0
87. 0
233. 1
131. 0
71. 1
173. 1
142. 0
Counts vs. 质荷比 (m/z)

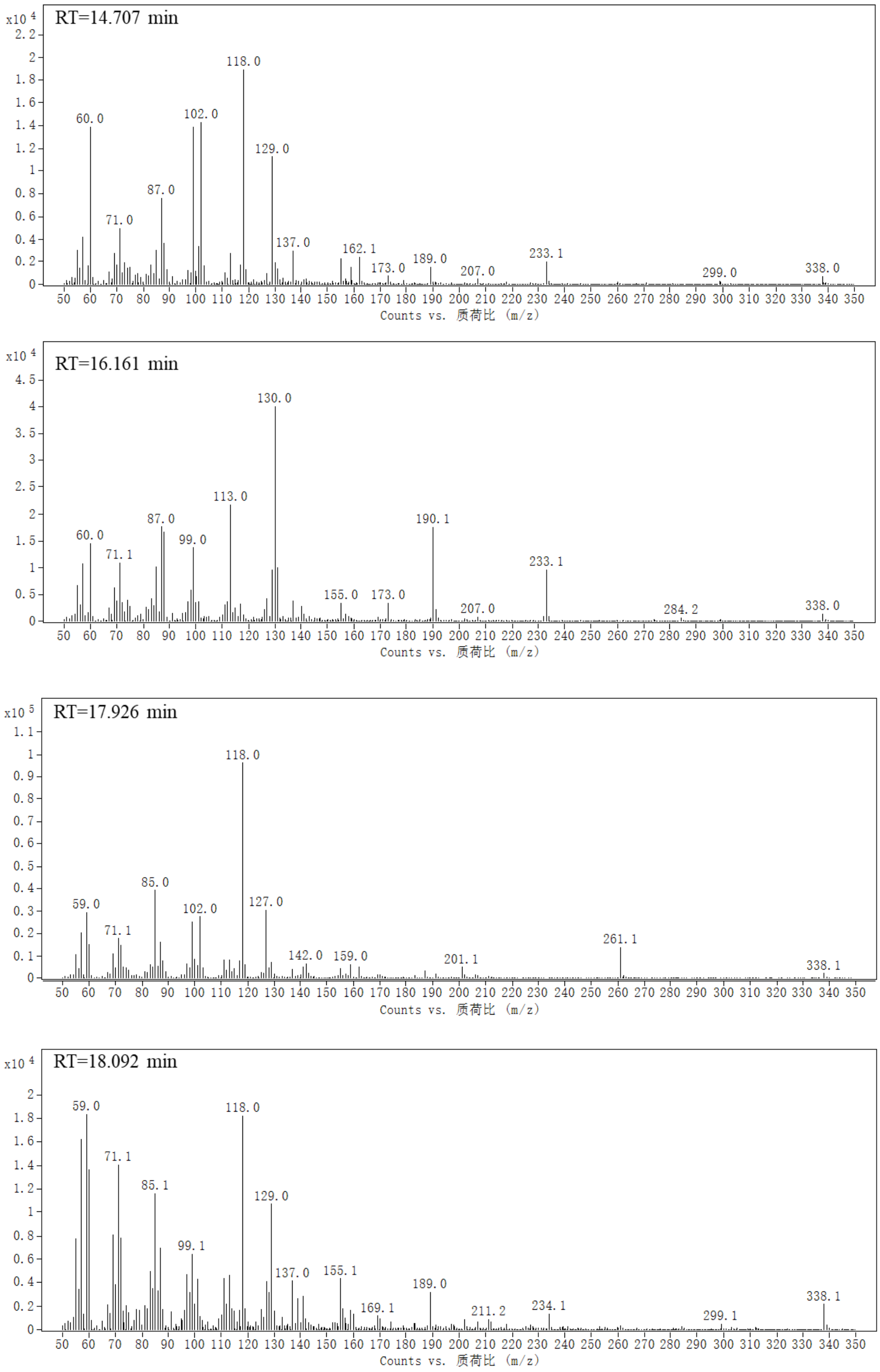

Figure S2. Tandem mass spectra of the characteristic peaks.

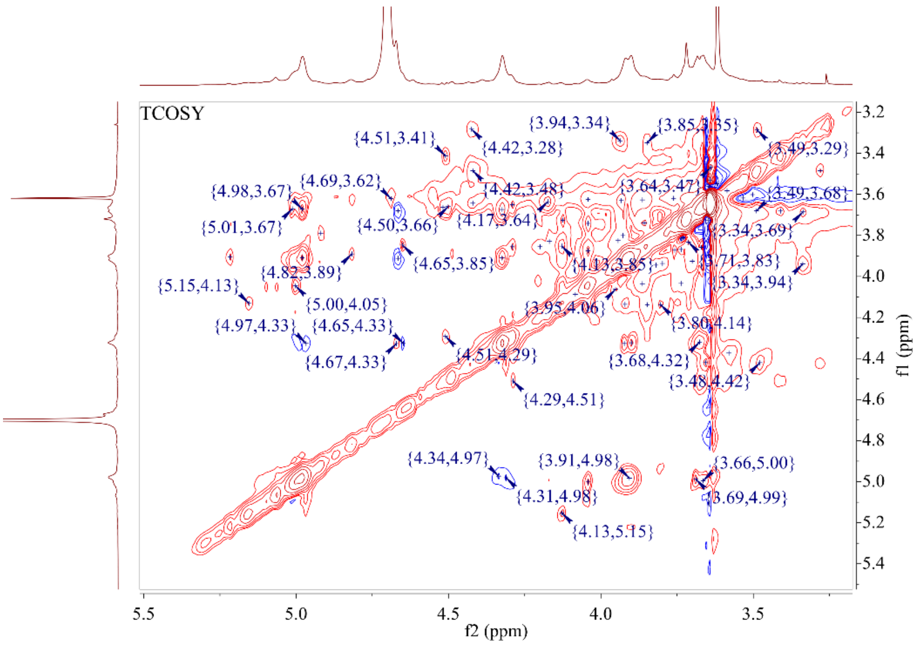

Figure S3. TOCSY spectra of RLPa-2 glycosyl residues.

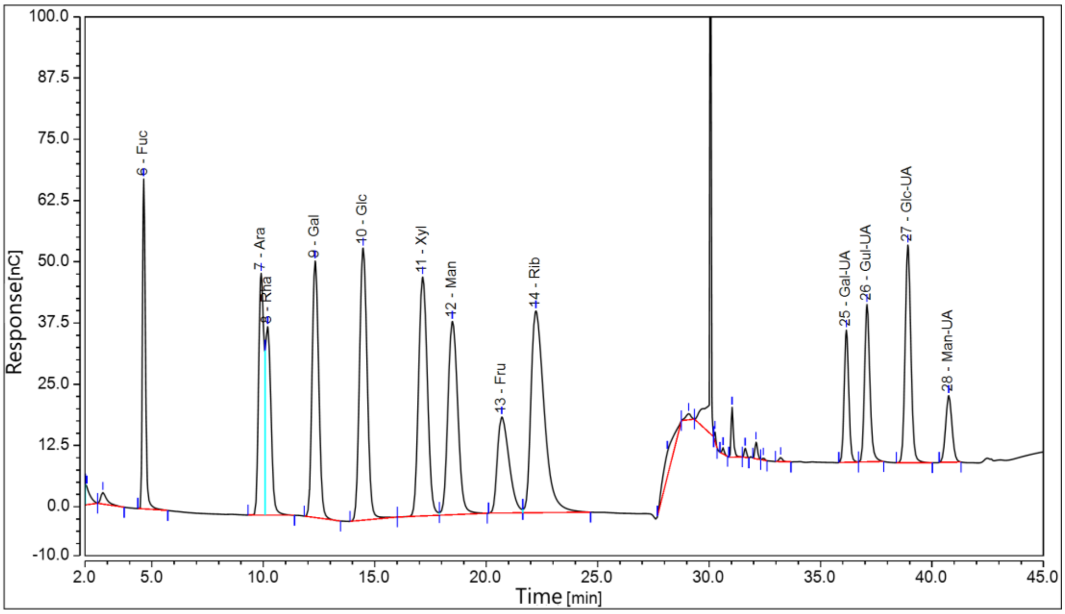

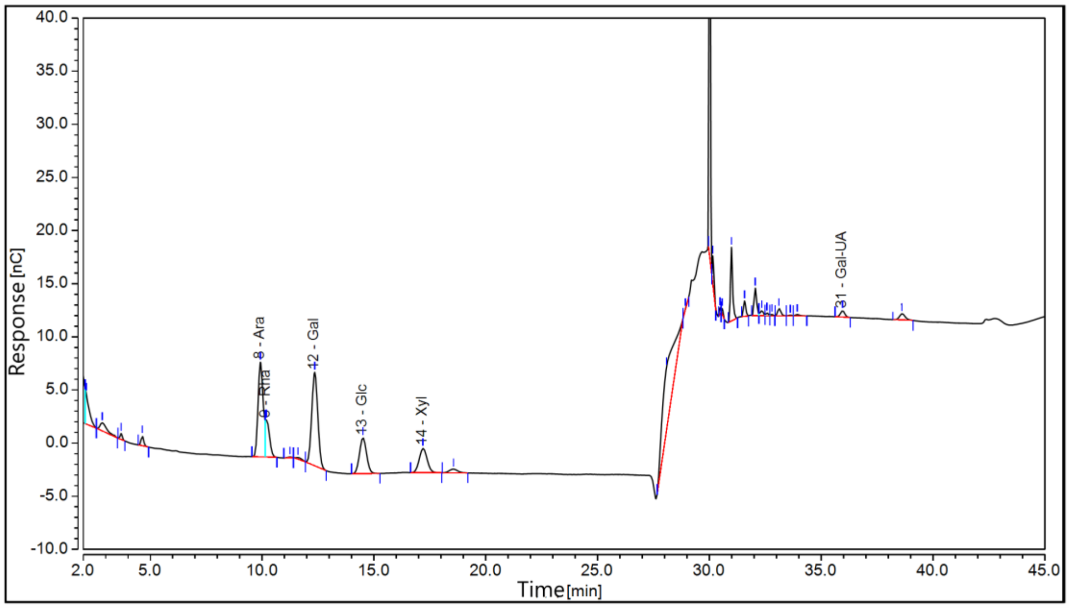

Figure S4. Chromatogram of monosaccharide standards and monosaccharide components in RLPa-2.

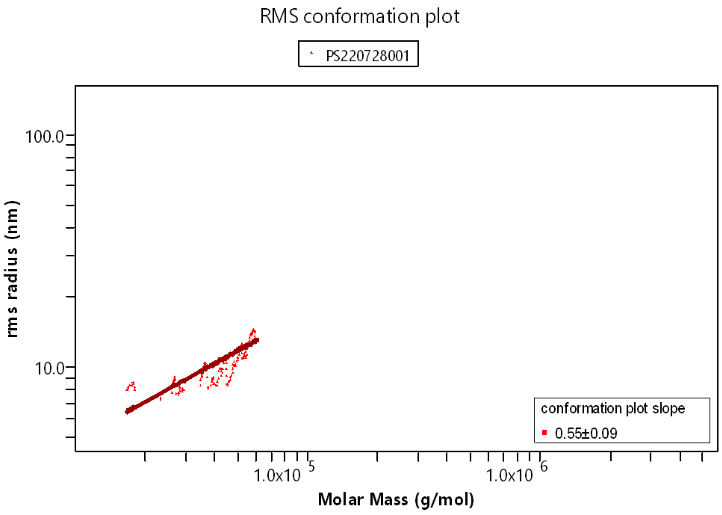

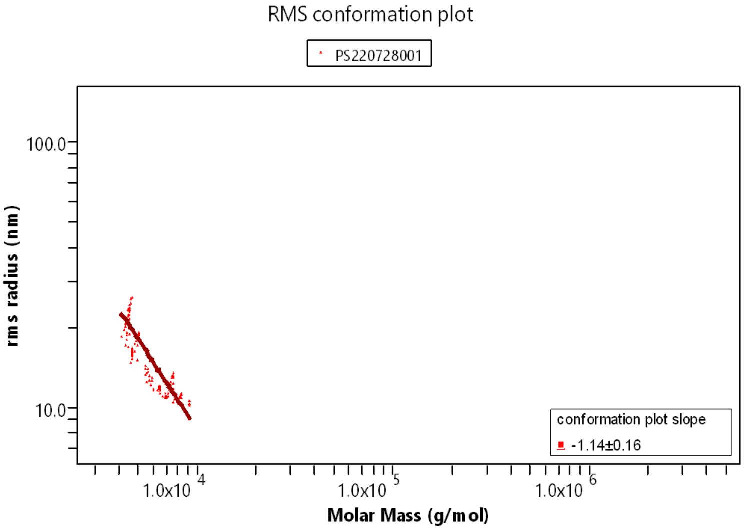

Figure S5. Molecular conformation diagram with different molar masses.

Table S1. The molecular parameters of RLPa-2 determined by SEC-MALLS-RI.

**Molecular Characteristics**	**Parameter**	**Detection Results**	**Uncertainty**
Molar mass moments	M_n	8.9 kDa	0.02709
	M_p	9.0 kDa	0.01535
	M_w	15.6 kDa	0.01387
	M_z	29.6 kDa	0.02933
	Polydispersity *(Mw/Mn)*	1.7	0.03043
Rms radius moments	R_n	12.3 nm	0.593
	R_w	10.6 nm	0.572
	R_z	10.3 nm	0.425
